# Clinical and Genetic Characteristics of Chinese Children With GLUT1 Deficiency Syndrome: Case Report and Literature Review

**DOI:** 10.3389/fgene.2021.734481

**Published:** 2021-11-22

**Authors:** Qingqing Hu, Yuechi Shen, Tangfeng Su, Yan Liu, Sanqing Xu

**Affiliations:** ^1^ Department of Pediatrics, Tongji Hospital, Tongji Medical College, Huazhong University of Science and Technology, Wuhan, China; ^2^ Department of Pediatrics, The First Affiliated Hospital of Nanchang University, Nanchang, China

**Keywords:** GLUT1 deficiency syndrome, epilepsy, developmental delay, movement disorders, SLC2A1 gene

## Abstract

**Objective**: GLUT1 deficiency syndrome (GLUT1-DS) is a rare, treatable neurometabolic disorder. However, its diagnosis may be challenging due to the various and evolving phenotypes. Here we report the first Chinese familial cases with genetically confirmed GLUT1-DS and analyze the characteristics of Chinese children with GLUT1-DS from clinical, laboratory, and genetic aspects.

**Methods**: We reported a Chinese family with three members affected with GLUT1-DS and searched for relevant articles up to September 2020 from PubMed, WOS, CNKI, and WanFang databases. A total of 30 Chinese patients diagnosed with GLUT1-DS (three newly identified patients in one family and 27 previously reported ones) were included and analyzed in this study.

**Results**: The median age of onset of the 30 patients (male: 18, female: 12) was 8.5 months (range, 33 days to 10 years). Epileptic seizures were found in 25 patients, most with generalized tonic–clonic and focal ones. Movement disorders were found in 20 patients—frequently with ataxia and dystonia, developmental delay in 25 patients, and microcephaly only in six patients. The cerebrospinal fluid (CSF) analysis showed decreased CSF glucose (median: 1.63 mmol/L, range: 1.1–2.6 mmol/L) and glucose ratio of CSF to blood (median: 0.340; range: 0.215–0.484). The genetic testing performed in 28 patients revealed 27 cases with pathogenic variations of the SLC2A1 gene, including 10 missense, nine frameshift, three nonsense, three large fragment deletions, and two splice-site mutations. Most patients had a good response to the treatment of ketogenic diet or regular diet with increased frequency. Although three patients in this Chinese family carried the same pathogenic mutation c.73C > T (p.Q25X) in the SLC2A1 gene, their symptoms and responses to treatment were not exactly the same.

**Conclusion**: The clinical manifestations of GLUT1-DS are heterogeneous, even among family members sharing the same mutation. For children with unexplained epileptic seizures, developmental delay, and complex movement disorders, detection of low CSF glucose or SLC2A1 gene mutations is helpful for the diagnosis of GLUT1-DS. Early initiation of ketogenic diet treatment significantly improves the symptoms and prognosis of GLUT1-DS.

## Introduction

Glucose transporter type 1 deficiency syndrome (GLUT1-DS) is caused by impaired glucose transport through the blood–brain barrier and inherited in an autosomal dominant trait, which results from mutations of SLC2A1 gene encoding GLUT1 (Brockmann et al., 2001; Klepper et al., 2001). Autosomal recessive transmission has also been described in rare cases, whereas homozygosity showed embryonic lethality in a mouse model (Wang et al., 2006; Klepper et al., 2009; Rotstein et al., 2010). The classic phenotype is characterized by refractory infantile epilepsy, developmental delay, acquired microcephaly, and complex movement disorders, such as ataxia, dystonia, and spasticity, which was first described by De Vivo *et al*. in 1991 (De Vivo et al., 1991). Over the past few decades, its clinical spectrum has broadened to include non-classic phenotypes, such as paroxysmal exercise-induced dyskinesia and epilepsy, paroxysmal choreoathetosis with spasticity, atypical childhood absence epilepsy, and myoclonic astatic epilepsy (Wang et al., 2018). Additionally, paroxysmal non-epileptic events, including intermittent ataxia, paroxysmal eyeball movements, dysarthria, migraine, alternating hemiplegia, spastic paraparesis, and periodic confusion (De Giorgis and Veggiotti, 2013; Pearson et al., 2013; Nicita et al., 2019), and some extraneurologic features, like hemolytic anemia and cataracts, have also been described (Weber et al., 2008; Flatt et al., 2011; Bawazir et al., 2012). Despite its complex and variable clinical manifestations, GLUT1-DS can be diagnosed based on the presence of hypoglycorrhachia and pathogenic variants of SLC2A1 gene. Ketogenic diet (KD) is recognized as the most effective treatment, which can provide ketone bodies to serve as an alternative fuel for the brain, and the earlier it is used, the better the prognosis will be (Kass et al., 2016).

In 2008, Bao *et al*. diagnosed a Chinese GLUT1-DS patient for the first time in mainland China and then reported three Chinese GLUT1-DS ones in 2012 (Liu et al., 2012). However, most (90%) detected SCL2A1 gene mutations are sporadic and *de novo*, whereas familial cases are rare (Pearson et al., 2013). Here we reported a Chinese family with three members affected with genetically confirmed GLUT1-DS and evaluated the efficacy of KD. Meanwhile, we analyzed and summarized the clinical and genetic characteristics of GLUT1-DS in the newly identified and previously reported Chinese GLUT1-DS cases.

## Patients and Methods

### Case Presentation

The clinical data and blood and cerebrospinal fluid (CSF) samples of three GLUT1-DS patients from one Chinese family were collected at the pediatric neurology clinics of Tongji Hospital. The study was approved by the Medical Ethics Committee of Tongji Hospital, Tongji Medical College, Huazhong University of Science and Technology. Informed consent was obtained from the patients and their parents.

#### Patient 1

The proband was a 6-year-old girl born to non-consanguineous parents. The pregnancy and the delivery were normal. She began to speak and walk independently at the age of 19 months and had a normal head circumference. The seizures occurred at the age of 8 months and 1 year, respectively, presenting with eyes rolling involuntarily and lips cyanosis, but there were no seizures in the next 2 years. Until the age of 4 years, her symptoms evolved into dystonia, majorly with the head backward, stiffness of the right lower limbs, and hallux dorsal flexion. Occasionally, her right arm was rotated inward and could not be flexed, accompanied by crying and shaking of the whole body. At the early stage, these symptoms were mild, with a frequency of three to eight times per month, each lasting about 1 to 2 min. The electroencephalogram (EEG) showed sharp waves and sharp slow waves in the right central temporal and left central region with a normal background during wake and sleep. Oral oxcarbazepine was then administered, but the attacks were not controlled completely. Since the age of 5 years, the frequency and duration of the attacks increased, ranging from once every 2 to 3 days to twice to thrice a day and lasting up to half an hour each time. Additionally, she experienced frequent paroxysmal hypokinesia with low muscle tone, which were relieved after resting. Nervous system physical examination was performed during the interictal period, and there was no abnormal muscle tension or strength and no positive ataxia signs, while the Assessment of the Child Development Scale indicated that her development was lagging behind mildly. The metabolic screening and other blood examinations were normal. KD was introduced along with oral oxcarbazepine when she was 5 years old, and it was tolerated well during the follow-up. Up to now, she has been free of these symptoms for nearly a year. The EEG returned to normal after initiating KD for 2 months.

#### Patient 2

The older brother of the proband was a 9-year-old boy without microcephaly. He started speaking after more than 12 months and still walked unsteadily at the age of 2 years and easily fell down. He also had paroxysmal hypokinesia which occurred about once to twice a month and lasted 1–5 min each time. At 3 years old, he had binocular gaze in a daze with clear consciousness, similar to an atypical absence seizure. His physical examination was unremarkable, and the developmental assessment showed that his development was lagging behind mildly as well. His brain magnetic resonance imaging (MRI), video EEGs, serum biochemistry values, and metabolic screening results were all normal. Considering the possible diagnosis of paroxysmal events related to epilepsy, sodium valproate (VPA) was added. He displayed some responses to VPA, with the symptoms alleviated only partly. Then, he received CSF analysis and gene panel sequencing, which indicated hypoglycorrhachia (CSF glucose: 2.6 mmol/L; CSF/blood glucose ratio: 0.481) and a pathogenic mutation in the SLC2A1 gene c.73C > T (p.Q25X). All these results clarified the final diagnosis of “GLUT1-DS,” but his family did not pay attention to it further. His symptoms worsened at the age of 6 years when VPA was discontinued by his parents, accompanied with an inability to speak or speaking vaguely—also called dysarthria and attacks of hallux-dorsiflexion, which lasted for a few minutes each time and were relieved spontaneously. Therefore, KD was introduced, and his symptoms remitted within 1 month. Up to now, he could go to school normally.

#### Patient 3

The father of patients 1 and 2, now at 31 years old, began to experience paroxysmal hypokinesia and could not move or walk for up to 10 min every time since he was 10 years old, without typical epileptic seizures. Surprisingly, although without any therapy, his symptom disappeared about 2 years ago. All these data were obtained from his description but no adult nervous physical examinations have been conducted.

#### Family History

**FIGURE 1:**
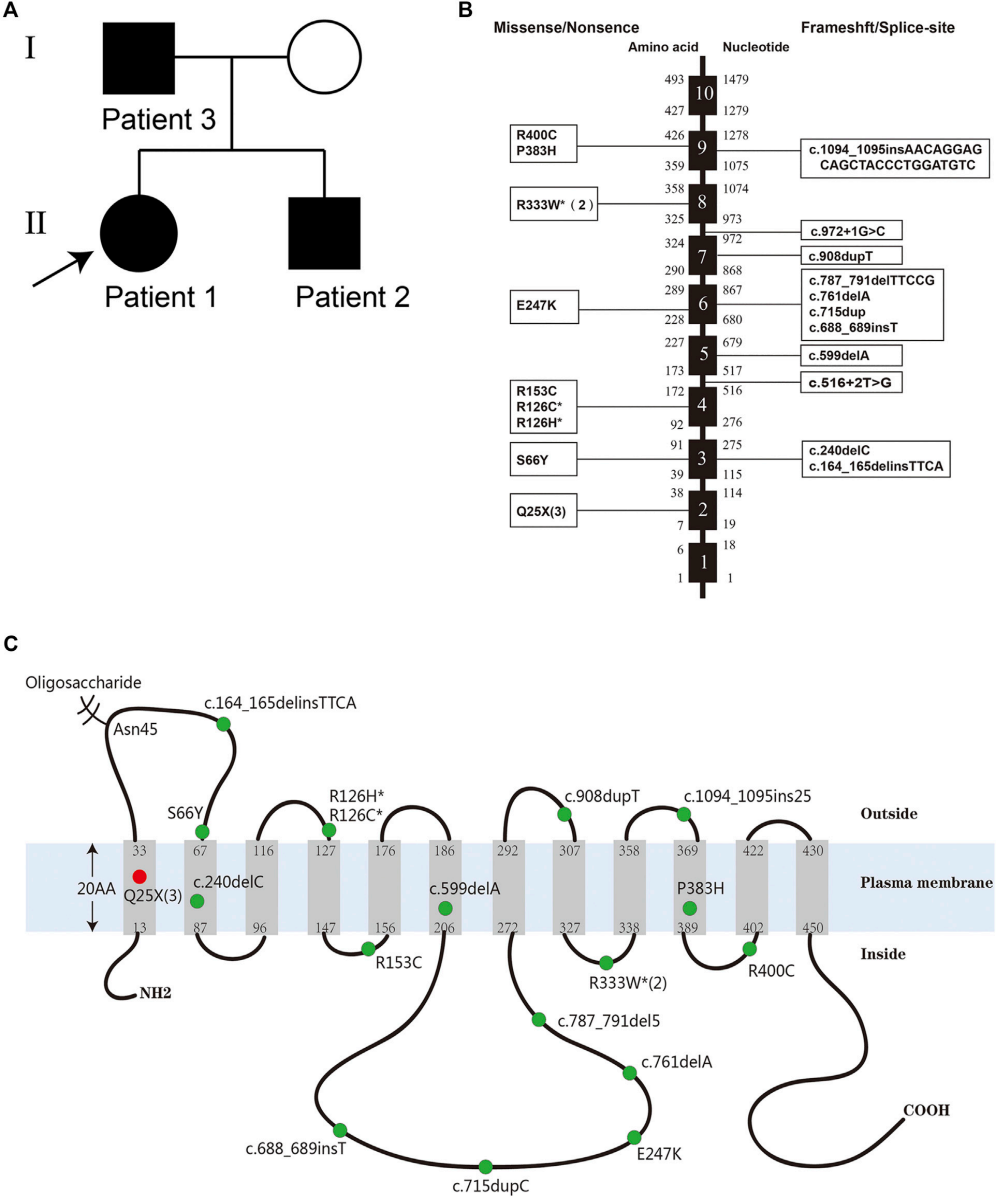
**(A)** The pedigree of the reported family with GLUT1-DS caused by a nonsense mutation c.73C > T (p.Q25X) in SLC2A1 gene. **(B)** Distribution of the reported mutations of SLC2A1 gene. The black boxes represent exons. The numbers to the left and the right of the intron–exon boundaries represent amino acid residues and nucleotides, respectively. The three large fragment deletion mutations and one missense mutation with limited information are not listed. **(C)** The conformational model of GLUT1 and the location of the reported mutations of the SLC2A1 gene. The three large fragment deletions and two splice-site mutations are not listed. The red dot represents a novel mutation c.73C > T (p.Gln25*) found in the familial cases. Some numbers between brackets represent the total number of patients identified with the mutation. Mutation hotspots are represented with an asterisk.

The pedigree is shown in Figure 1A. The uncle of patient 3 had similar attacks of movement disorder, but his information was incomplete and unavailable.

### Genetic Analyses and *In Silico* Analyses

Whole-exome sequencing was performed in the proband (patient 1), and it revealed the pathogenic mutation c.73C > T (p.Q25X) in the SLC2A1 gene, which was identical to that of patient 2. The mutation was confirmed by Sanger sequencing in their parents and verified to be inherited from their father (patient 3). The nonsense variant was predicted by PVS1 as pathogenic based on the guidelines of the American College of Medical Genetics and Genomics.

The heterozygous substitution c.73C > T (p.Q25X) is a novel nonsense mutation in exon 2 that has not been previously reported, which is predicted to cause a premature termination at position 25 and the deletion of 467 amino acids (Figures 1B,C). The truncated protein contains only less than one of the wild type 12 transmembrane domains and easily degrades in the cell. A previous study has shown that, when the last 37 carboxyl-terminal amino acids of GLUT1 were lost, the truncated proteins would abolish transport activity completely (Oka et al., 1990).

### Systematic Review

We searched literatures about Chinese case reports of GLUT1-DS in PubMed, WOS, CNKI, and WanFang databases (until September 2020). Patients who were described more than once in the literatures were included only one time. Based on the systematic review, 15 articles describing 27 Chinese patients with GLUT1-DS were found (Fung et al., 2011; Liu et al., 2012; Ye et al., 2012; Liu et al., 2013; Li et al., 2014; Yu and Long, 2015; Duan et al., 2016; Zhang et al., 2017; Ji et al., 2018; Liang et al., 2019; Wang et al., 2019; Wei et al., 2019; Zha et al., 2019; Pei et al., 2020; Wang et al., 2020). Thus, together with the three newly identified familial cases, a total of 30 Chinese patients diagnosed with GLUT1-DS were included in this study.

## Results

Among the 30 patients, there were 18 males and 12 females. The median age of onset was 8.5 months (range, 33 days to 10 years). Epileptic seizures were reported in 25 patients (83.3%), presenting as the first symptom in 23 patients (76.7%) during infancy. The seizures were of mixed types, including 11 cases of generalized tonic–clonic (GTC) (36.7%), nine cases of focal (30%), four cases of tonic (13.3%), three cases of absence (10%), two cases of atonic (6.7%), one case of spasm (3.3%), one case of myoclonic–atonic (3.3%), and one case of febrile seizures (3.3%). Most seizures were resistant to antiseizure medications (ASMs), such as phenobarbital, valproic acid, lamotrigine, levetiracetam, and oxcarbazepine. Movement disorders were reported in 20 patients (66.7%), including ataxia in 15 cases (50.0%), dystonia in 14 cases (46.7%), gait disturbance in eight cases (26.7%), dysarthria in seven cases (23.3%), paroxysmal hypokinesia in three cases (10.0%), tremor in two cases (6.7%), paroxysmal limb paralysis in two cases (6.7%), and paroxysmal eye movement disorder in two cases (6.7%). Developmental delay was observed in 25 patients (83.3%), and microcephaly was found only in six patients (20%). The clinical characteristics of 30 Chinese patients with GLUT1-DS are summarized in Table 1.

**TABLE 1:** Clinical characteristics of 30 Chinese patients with GLUT1-DS identified from 2008 to 2020.

Pt	Sex/age at onset	Seizure type	MD	DD	MC	CSF glucose (mmol/L)	CSF/blood glucose ratio	EEG	Brain MRI/CT	Genetic testing	Treatment (response)	Ref
1	M/18m	Spasm#	Ataxia, dystonia	+	+	1.80	0.382	-	-	+	KD (+)	Fung et al. (2011)
2	F/8m	Absence#	Ataxia	+	-	1.90	0.387	-	-	+	Counseling for MAD	Fung et al. (2011)
3	M/2m	GTCS#	-	+	-	1.10	0.215	δ activity	-	N/A	KD (+)	Ye et al. (2012)
4	M/41d	Focal#, tonic	-	+	-	low	Low	Epileptiform discharge	Mild brain atrophy	+	KD (+)	Liu et al. (2013)
5	M/60d	Focal#	-	+	-	low	Low	-	-	+	KD (+)	Liu et al. (2013)
6	M/33d	Focal#, tonic	Gait disturbance, ataxia	+	-	low	Low	-	Bilateral ventricle plump	+	Increased frequency of regular diet (+)	Liu et al. (2013)
7	F/1y	-	Ataxia#, dysarthria, gait disturbance, dystonia	+	-	1.63	0.402	-	Mild hypomyelination	+	KD (intolerance)	Liu et al. (2012)
8	M/5y9m	-	Gait disturbance #, ataxia#, dystonia	-	-	2.40	0.484	Epileptiform discharge	-	+	Increased frequency of regular diet (+)	Liu et al. (2012)
9	F/2y	Focal	Gait disturbance #, ataxia#, dystonia	+	+	1.74	0.375	-	-	+	Increased frequency of regular diet (+)	Liu et al. (2012)
10	F/6m	GTCS#	Dystonia	+	+	1.60	0.240	Background slowing	N/A	-	KD (intolerance)	Li et al. (2014)
11	F/73d	Focal#	-	+	-	2.10	0.340	Epileptiform discharge, background slowing	N/A	+	Increased frequency of regular diet (+-); KD is planned	Li et al. (2014)
12	M/2m	GTCS#	Ataxia	+	-	1.50	0.319	Background slowing	-	+	KD (+)	Yu and Long, (2015)
13	M/9m	GTCS#	Ataxia	-	+	1.87	0.368	Epileptiform discharge	-	+	N/A	Duan et al. (2016)
14	F/1y6m	-	Dystonia#, ataxia#, gait disturbance	+	+	N/A	N/A	N/A	White matter dysplasia	+	KD (+)	Zhang et al. (2017)
15	F/11m	Tonic#, Atonic	Ataxia, dystonia, dysarthria, tremor, paroxysmal limb paralysis	+	-	low	Low	Epileptiform discharge	-	+	KD (+)	Ji et al. (2018)
16	M/15m	GTCS#, Atonic, Focal	Ataxia, dystonia, dysarthria, paroxysmal limb paralysis and eye movement disorders	+	-	low	Low	Epileptiform discharge	Abnormal signals in bilateral frontal cortex	+	KD (+)	Ji et al. (2018)
17	M/2m	GTCS#	Ataxia, dystonia, dysarthria, tremor, paroxysmal eye movement disorders	+	-	low	Low	Epileptiform discharge	Widened bilateral frontotemporal sulci	+	KD (+)	Ji et al. (2018)
18	F/6m	GTCS#	Ataxia, dystonia, dysarthria	+	-	low	Low	Few atypical sharp waves	-	+	KD (+)	Ji et al. (2018)
19	M/2m	Focal#, GTCS	-	+	+,-	1.22	0.312	Epileptiform discharge	-	+	KD (+)	Wang et al. (2019)
20	F/2y7m	FS#, Absence	-	-	-	1.62	0.337	Epileptiform discharge	-	+	KD (+)	Liang et al. (2019)
21	M/3y7m	-	Ataxia, dystonia, gait disturbance dysarthria	+#	-	N/A	N/A	-	-	+	KD (+)	Liang et al. (2019)
22	F/1y3m	GTCS#	-	+	-	N/A	N/A	Epileptiform discharge	Cerebral dysplasia	+	KD (+)	Liang et al. (2019)
23	M/1y	MAE#	Gait disturbance	+	-	N/A	N/A	Epileptiform discharge	N/A	+	KD (+)	Wei et al. (2019)
24	M/45d	GTCS#	-	+	-	1.33	0.246	-	-	+	KD (+)	Zha et al. (2019)
25	F/65d	GTCS#	Ataxia, dystonia	+	-	1.43	0.332	Background slowing	Hypomyelination	N/A	KD (+)	Zha et al. (2019)
26	M/6y8m	Focal#	-	-	-	1.83	0.373	Borderline	Arachnoid cyst, hippocampal sclerosis	+	KD (+)	Pei et al. (2020)
27	M/2m	Tonic #	-	+	-	1.38	0.238	Epileptiform discharge	-	+	KD (+)	Wang et al. (2020)
28	F/8m	Focal#	Dystonia, paroxysmal hypokinesia	+	-	N/A	N/A	Epileptiform discharge	N/A	+	KD (+)	This study
29	M/2y	Absence	Gait disturbance #, paroxysmal hypokinesia #, dystonia, dysarthria	+	-	2.60	0.481	-	-	+	KD (+)	This study
30	M/10y	-	Paroxysmal hypokinesia #	-	-	N/A	N/A	N/A	N/A	+	N/A	This study

Pt, patient; F, female; M, male; m, months; y, years; d, days; MD, movement disorder; DD, developmental delay; MC, microcephaly; GTCS, generalized tonic–clonic seizure; FS, febrile seizures; MAE, myoclonic–atonic epilepsy; CSF, cerebrospinal fluid; KD, ketogenic diet; MAD, modified Atkins diet; N/A, not available; +, positive; -, negative; #, initial symptoms.

A fasting lumber puncture was performed in 24 patients, but only 17 patients reported definite values of CSF analysis, with low CSF glucose at 1.1–2.6 mmol/L (median, 1.63 mmol/L) and low CSF/blood glucose ratio of 0.215–0.484 (median, 0.340).

EEG was performed in 28 of 30 patients, showing generalized or focal epileptiform discharges or background slowing in 19 cases. No abnormalities were found in the remaining nine cases. Brain MRI was performed in 25 patients. Only nine cases exhibited abnormalities, such as mild brain atrophy, bilateral ventricle enlargement, hypomyelination, abnormal signals in the bilateral frontal cortex, widening of the bilateral frontotemporal sulci, and cerebral dysplasia.

Genetic testing was performed in 28 patients. Except for one patient who was negative for SLC2A1 gene mutation, 27 patients carried pathogenic mutations in the SLC2A1 gene, including 10 with missense, nine with frameshift, three with nonsense, three with large fragment deletion, and two with splice-site mutations (Table 2).

**TABLE 2:** Twenty-four different mutations of the SLC2A1 gene in 27 Chinese patients with GLUT1-DS.

Mutation	Pt.	Exon	Nucleotide	Location	Mutation origin
Missense					
R333W	2, 22	8	c. 997C>T	Cytoplasmic loop 8–9	*De novo*
E247K	5	6	c.741G > A	Cytoplasmic loop 6–7	*De novo*
P383H	8	9	c.1148C > A	TMD10	*De novo*
R400C	9	9	c.1198C > T	Cytoplasmic loop 10–11	*De novo*
N/A	14#	8	N/A	N/A	*De novo*
R126C	16	4	376G > A	Extracellular loop 3–4	*De novo*
R126H	17	4	377G > A	Extracellular loop 3–4	*De novo*
S66Y	19	3	197C > A	Extracellular loop 1–2	*De novo*
R153C	20	4	457C>T	Cytoplasmic loop 4–5	*De novo*
Nonsense					
Q25X	28–30	2	73C > T	TMD1	Paternal
Frameshift					
c.240delC	1	2	240delC	TMD2	N/A
c.599delA	6	5	599delA	TMD6	*De novo*
c.761delA	7	6	761delA	Cytoplasmic loop 6–7	N/A
c.787_791del5	15	6	787_791del TTCCG	Cytoplasmic loop 6–7	*De novo*
c.688_689insT	18	6	688_689insT	Cytoplasmic loop 6–7	*De novo*
c.715dupC	21	6	715dupC	Cytoplasmic loop 6–7	*De novo*
c.1094_1095ins25	23	9	1094_1095insAACAGGAGC AGCTACCCTGGATGTC	Extracellular loop 9–10	*De novo*
c.908dupT	26	7	908dupT	Extracellular loop 7–8	N/A
c.164_165delinsTTCA	27	3	164_165delinsTTCA	Extracellular loop 1–2	*De novo*
Large fragment deletion					
Large fragment deletion	4	2	exon 2 large fragment deletion	-	*De novo*
c.350_385del	13	4	350_385del	-	*De novo*
Large fragment deletion	24#	N/A	chr1:43392702–43409002	-	*De novo*
Splice site		Intron			
c.972+1G > C	11	7	972+1G > C	-	N/A
c.516+2T > G	12	4	516+2T > G	-	*De novo*

Two patients (Pt. 14 and Pt. 24) only showed limited information of mutations. One patient (Pt. 10) negative for SLC2A1 gene mutation and two (Pt. 3 and Pt. 25) without available mutation data were not presented here.

Twenty-three patients were treated with KD, 21 of whom became free from seizures and movement disorders. Two cases gave up KD treatment for intolerance, and their symptoms were not improved. Besides this, four patients were given a regular diet with increased frequency or advised to eat candy, three of whom had a significant improvement in their outcomes. The other three patients refused any treatment for their mild symptoms or were still counseled for KD treatment.

## Discussion

Glucose, the most essential energy source for brain metabolism, enters into the brain facilitated by GLUT1. The cerebral metabolic rate for glucose was low at birth and then increased rapidly to reach adult level by 2 years old. At about 3 to 4 years old, it reaches a peak that exceeds adult level by over two folds, keeps up until 9 years of age, and then gradually decreases to adult rate again by the age of 20 years (Chugani et al., 1987; Chugani, 1998). Consequently, the seizures generally occur at 1–6 months after birth as the most common initial symptom of GLUT1-DS and gradually reduce in adolescence, whereas these rarely appear during the neonatal period, but cyanotic or apnea episodes and paroxysmal eyeball movements—named aberrant gaze saccades—may occur before seizures in infancy (Pearson et al., 2017), which are easily ignored or misdiagnosed as focal seizures. Among the 30 patients in our study, the earliest onset age was 33 days, and the median age was 8.5 months. About 76.7% of the patients presented epileptic seizures as the first symptom.

Approximately 90% of GLUT1-DS patients have seizures of various types, including GTC, absence, focal, myoclonic, atonic, tonic, and spasm seizures, in which GTC and absence seizures are the most common (Pong et al., 2012). Although seizures are often resistant to ASMs, some of which even inhibit the GLUT1 function *in vitro*, such as phenobarbital, valproate, chloral hydrate, and diazepam (Klepper et al., 1999; Klepper et al., 2003; Wong et al., 2005), there are still about 8% of patients who respond positively to them (Pong et al., 2012). In this study, 83.3% of the 30 patients had epileptic seizures, and the seizure types in some patients evolved with age, with GTC (36.7%) and focal (30%) seizures mostly observed. Some patients achieved partial relief of seizures and motor disorders by taking ASMs, such as oxcarbazepine and levetiracetam. However, the epileptic seizures in such GLUT1-DS patients are not closely related to the epileptiform discharges on EEG. Our study showed that one patient without seizures was found to be with epileptiform discharges, while seven cases with seizures had normal EEG findings.

Compared with epileptic seizures, movement disorders are more common in older children and tend to replace seizures over the years (Leen et al., 2014). In this study, 66.7% of the 30 patients had movement disorders, with ataxia (50.0%), dystonia (46.7%), gait disturbance (26.7%), and dysarthria (23.3%) being more commonly observed.

Since infancy, the developing brain of GLUT1-DS patients is unable to obtain enough energy for its growth, leading to cerebral dysfunction and limited head growth. The majority of patients with GLUT1-DS have experienced some degree of cognitive impairment, ranging from mild learning disability to severe intellectual impairment. In this study, developmental delay was observed in 83.3% of the 30 patients, while microcephaly was not a key sign, only observed in 20% of patients. Furthermore, one patient with microcephaly had no developmental delay, indicating that microcephaly was not always accompanied by developmental delay.

The laboratory hallmark for GLUT1-DS is low glucose of the CSF (hypoglycorrhachia) when normoglycemic. Generally, fasting CSF glucose values are less than 3.3 mmol/L (60 mg/dl). In the majority (>90%) of cases, the values are less than 2.2 mmol/L (40 mg/dl). Another less reliable biomarker is the glucose ratio of CSF to blood below 0.45 (De Vivo and Wang, 2008; Yang et al., 2011; Klepper, 2012; Leen et al., 2013), but normal CSF glucose has also been reported in some patients with milder phenotypes. In addition, the CSF lactate level from normal to low (<1.4 mmol/L) can help differentiate GLUT1-DS from other causes of hypoglycorrhachia, such as meningitis, encephalitis, subarachnoidal haemorrhage, and some metabolic disorders, in which the CSF lactate level is elevated (>2.0 mmol/L) (Chow, 2005). Leen et al. (2013) suggested that it was unlikely to diagnose GLUT1-DS if the CSF lactate was increased. The CSF glucose and CSF/blood glucose ratio were low in this study, ranging 1.1–2.6 mmol/L (median 1.63 mmol/L) and 0.215–0.484 (median 0.340), respectively, which was consistent with that described above.

To date, SLC2A1 is recognized as the only gene associated with GLUT1-DS mapped to the short arm of chromosome 1 (1p34.2), with a length of about 35 kb and containing 10 exons (Shows et al., 1987; Fukumoto et al., 1988). Its coded protein GLUT1 is a transmembrane glycoprotein and composed of 492 amino acids. It has 12 transmembrane domains that span the plasma membrane in an α-helical structure, forming a pore for the transport of glucose and other substrates, and is mainly distributed on brain endothelial cells, astrocytes, and erythrocytes (Mueckler et al., 1985). Since the first elucidation of its genetic–pathogenic roles (Seidner et al., 1998), more than 200 SLC2A1 gene mutations have been described, including missense, nonsense, frameshift, splice-site, and large fragment deletion mutations. Several mutation hotspots within the SLC2A1 gene have been identified, such as Asn34, Gly91, Ser113, Arg126, Arg153, Arg264, Thr295, and Arg333 (Leen et al., 2010). However, about 5–10% of GLUT1-DS cases were reported with absence of mutations in the SLC2A1 gene (Yang et al., 2011; Klepper, 2013). In our study, there was one case with a negative SLC2A1 gene mutation as well. Therefore, some studies have suggested that GLUT1-DS may have undetectable variants in the non-coding regions or downstream of the DNA that could affect posttranscriptional modifications, such as mRNA splicing, protein assembly, folding, transportation, intracellular storage, and activation (Klepper and Leiendecker, 2007).

Furthermore, the phenotypes and symptom severity of GLUT1 patients with the same mutations were heterogeneous even among affected family members sharing the same mutation, indicating that secondary genes (such as PURA gene) and other modifying proteins might be involved in glucose transport or that DNA variant regulatory elements of the wild-type allele may modulate the expression level of GLUT1 (Leen et al., 2010; Mayorga et al., 2018). Mayorga et al. (2018) reported a patient with a PURA gene mutation that had marked hypoglycorrhachia, overlapping the clinical findings with GLUT1-DS, though no variants were found on the SLC2A1 gene. By western blot assays, they confirmed a significantly reduced expression of GLUT1 in the peripheral blood cells of the patient compared to controls. Based on the known functions of PURA as a transcriptional and translational regulator, they proposed GLUT1 as a new PURA target, and mutations in PURA can decrease GLUT1 expression.

Though familial cases were usually correlated with milder manifestations compared to sporadic *de novo* cases (De Giorgis et al., 2015), attention should be paid to prenatal genetic counseling in familial cases because of phenotypic heterogeneity—even if a patient has very few and mild symptoms, and it is possible that offspring with the same mutation will be severely affected. Herein the familial cases in this study, who carried a nonsense mutation c.73C > T (p.Gln25*) and inherited in an autosomal dominant manner, presented mild to moderate clinical phenotypes with overlapping but variable symptoms. The clinical manifestations of the father of the proband were mild, with only episodic hypokinesia but no intelligence or language disorder, which might be related to the late onset of the disease, while the phenotypes of the proband and her brother were moderate, including three main symptoms, and even evolved with age.

KD is the gold standard treatment for GLUT1-DS, which should be started as early as possible and maintained at least until adolescence to meet the increased energy demand of the developing brain (Klepper, 2012; Kass et al., 2016). In our study, most patients were free of seizures and movement disorders after initiation of KD, and intelligence development and the head circumference of some patients recovered to normal levels. The other three patients treated with a regular diet of increased frequency also showed an improvement that might be classified as “carbohydrate-responsive” phenotype.

In conclusion, the diagnosis of GLUT1-DS is challenging due to its clinical heterogeneity. When children presented with unexplained epileptic seizures, developmental delay, and complicated movement disorder, GLUT1-DS should be suspected and a fasting lumbar puncture or genetic testing is recommended. Once the diagnosis of GLUT1-DS is confirmed, KD should be administered earlier and timely in order to gain a better prognosis.

## Data Availability

The datasets for this article are not publicly available due to concerns regarding participant/patient anonymity. Requests to access the datasets should be directed to the corresponding author.
